# Metagenome-Assembled Genome Sequence of Marine *Rhizobiaceae* sp. Strain MnEN-MB40S, Obtained from Manganese-Oxidizing Enrichment Culture

**DOI:** 10.1128/mra.00645-22

**Published:** 2022-08-17

**Authors:** Masataka Aoki, Nozomi Nakahara, Masataka Kusube, Kazuaki Syutsubo

**Affiliations:** a Regional Environment Conservation Division, National Institute for Environmental Studies, Tsukuba, Ibaraki, Japan; b Department of Civil Engineering, National Institute of Technology, Wakayama College, Gobo, Wakayama, Japan; c Bioproduction Research Institute, National Institute of Advanced Industrial Science and Technology, Tsukuba, Ibaraki, Japan; d Department of Applied Chemistry and Biochemistry, National Institute of Technology, Wakayama College, Gobo, Wakayama, Japan; e Research Center for Water Environment Technology, School of Engineering, the University of Tokyo, Bunkyo, Tokyo, Japan; University of Southern California

## Abstract

Here, we report a new metagenome-assembled genome (MAG) from a marine *Rhizobiaceae* species. The MnEN-MB40S genome was assembled from a manganese-oxidizing enrichment culture metagenome. A 4.1-Mb MAG comprising 26 contigs, with a GC content of 60.0%, was obtained. This MAG contributes to the genomic information regarding the family *Rhizobiaceae*.

## ANNOUNCEMENT

The family *Rhizobiaceae* comprises genera that are mainly associated with soil and plant hosts (1). Some *Rhizobiaceae* genera can potentially be used for bioremediation of heavy metals and biodegradation of toxic compounds (1). However, their metabolic capabilities and ecological roles, particularly in marine environments, remain unclear because of the limited availability of cultured marine isolates and their sequenced genomes.

Here, we report a new *Rhizobiaceae*-associated metagenome-assembled genome (MAG) that was derived from a marine manganese-oxidizing enrichment culture originating from seawater (water depth, 0 m) in the Nada coastal area of Wakayama, Japan (33°49′52.6″N, 135°10′31.8″E) (2). Briefly, seawater (100 mL), used as an enrichment inoculum, was collected directly in a 500-mL glass bottle. A 1-L-scale enrichment experiment was performed using polycaprolactone (as a solid organic substrate) and a MARINE ART SF-1 artificial seawater (Osaka Yakken Co., Ltd.)-based medium under aerobic conditions at ~25°C (see reference 2 for details). After 3,340 h of enrichment, 22.5 mL of the planktonic fraction of the enrichment culture was collected using a 25-mL pipette and stored as a glycerol stock at −80°C until DNA extraction. Total genomic DNA for metagenomic sequencing was extracted using an MPure bacterial DNA extraction kit (MP Biomedicals). A sequencing library was prepared using an MGIEasy FS DNA library prep set (MGI Tech Co., Ltd.) and an MGIEasy DNA adapters-96 (plate) kit (MGI). Circularized DNA and DNA nanoballs were prepared using an MGIEasy circularization kit (MGI) and a DNBSEQ-G400RS high-throughput sequencing kit (MGI), respectively. Paired-end (2 × 200-bp) sequencing on a DNBSEQ-G400 sequencer (MGI) produced 135,388,150 raw sequence reads (total of 27,077,630,000 bp). Default parameters were used for all software unless otherwise specified. Adapter sequences were removed, and the raw sequence reads were quality filtered using fastp v0.20.0 (quality scores of ≥30) (3). Quality-filtered reads were assembled using MEGAHIT v1.2.9 (4). Assembled contigs were binned using MaxBin 2.0 v2.2.7 (5), MetaBAT 2 v2.12.1 (6), and MyCC vMyCC_2017 (7) and then refined using DAS Tool v1.1.3 (8). Genes were predicted using the DFAST pipeline v1.2.16 (https://dfast.ddbj.nig.ac.jp) (9). Genome coverage was calculated using Minimap2 v2.23 (10). The EzBioCloud 16S rRNA gene-based identification service (database v2021.07.07) (11) was used for the 16S rRNA gene similarity search. Taxonomic affiliation was determined using GTDB-Tk v1.7.0 and the Genome Taxonomy Database (GTDB) R202 (12) in KBase (13). MAG completeness and contamination were calculated using CheckM (marker set, *Alphaproteobacteria*) (14) in DFAST.

Table 1 summarizes the genome statistics and taxonomic information of *Rhizobiaceae* MAG MnEN-MB40S. The sequence similarity of the 16S rRNA gene to the closest cultivated relative (97.58%) was below the proposed species boundary (15). A BLASTp search (E value threshold, 1 × 10^−20^; query coverage, ≥70%; sequence identity, ≥40%) (16) against nonredundant sequences in NCBI and UniProtKB/Swiss-Prot revealed the presence of putative multicopper oxidase (MCO) genes [related to Mn(II) oxidation]. The locus tags MnENMB40S_08170 and MnENMB40S_10010 were homologous to the MCO gene *moxA* from *Pedomicrobium* sp. strain ACM 3067 (GenBank accession number CAJ19378) (71.8% sequence identity) (17) and the MCO gene *cueO* from Escherichia coli K-12 MG1655 (GenBank accession number P36649) (44.6% sequence identity) (18), respectively.

**TABLE 1:** Genome statistics and taxonomic information for *Rhizobiaceae* MAG MnEN-MB40S

Parameter	Finding
Genome size (bp)	4,129,535
No. of contigs	26
GC content (%)	60.0
*N*_50_ (bp)	294,856
Genome coverage (×)	59
No. of coding sequences	3,879
No. of rRNA genes (5S, 16S, 23S)	1 (0, 1, 0)
No. of tRNA genes	32
Completeness (%)	75.34
Contamination (%)	0.00
Taxonomic affiliation	
Phylum level	*Proteobacteria*
Class level	*Alphaproteobacteria*
Order level	*Rhizobiales*
Family level	*Rhizobiaceae*
Genus level	SPNT01
Closest cultivated relative based on 16S rRNA gene sequence similarity	*Hoeflea prorocentri* PM5-8
Similarity to closet cultivated relative (%)	97.58
GenBank accession no. for closet cultivated relative	KY264918

### Data availability.

The annotated MAG of *Rhizobiaceae* sp. strain MnEN-MB40S is available in DDBJ/EMBL-Bank/GenBank under the accession numbers BRLC01000001 to BRLC01000026. The raw DNBSeq read data have been deposited in the Sequence Read Archive (SRA) under the accession number DRX364682.
